# Insulin Dynamics in Young Women with Polycystic Ovary Syndrome and Normal Glucose Tolerance across Categories of Body Mass Index

**DOI:** 10.1371/journal.pone.0092995

**Published:** 2014-04-04

**Authors:** Melania Manco, Lidia Castagneto-Gissey, Eugenio Arrighi, Annamaria Carnicelli, Claudia Brufani, Rosa Luciano, Geltrude Mingrone

**Affiliations:** 1 Research Unit for Multi-factorial Diseases, Obesity and Diabetes, Scientific Directorate, Bambino Gesù Children Hospital, Rome, Italy; 2 Department of Internal Medicine, Catholic University, School of Medicine, Rome, Italy; University of Texas Health Science Center at San Antonio, United States of America

## Abstract

**Background:**

Evidence favours insulin resistance and compensatory hyperinsulinemia as the predominant, perhaps primary, defects in polycystic ovary syndrome (PCOS). The aim of the present study was to evaluate insulin metabolism in young women with PCOS but normal glucose tolerance as compared with age, body mass index and insulin resistance-matched controls to answer the question whether women with PCOS hypersecrete insulin in comparison to appropriately insulin resistance-matched controls.

**Research Design and Methods:**

Sixty-nine cases were divided according to their body mass index (BMI) in normal-weight (N = 29), overweight (N = 24) and obese patients (N = 16). Controls were 479 healthy women (age 16–49 y). Whole body Insulin Sensitivity (WBISI), fasting, and total insulin secretion were estimated following an oral glucose tolerance test (C-peptide deconvolution method).

**Results:**

Across classes of BMI, PCOS patients had greater insulin resistance than matched controls (p<0.0001 for all the comparisons), but they showed higher fasting and total insulin secretion than their age, BMI and insulin resistance-matched peers (p<0.0001 for all the comparisons).

**Conclusion:**

Women with PCOS show higher insulin resistance but also larger insulin secretion to maintain normal glucose homeostasis than age-, BMI- and insulin resistance-matched controls.

## Introduction

Polycystic ovary syndrome (PCOS) occurs in about 10% of women of reproductive age, thus representing one of the commonest endocrine disorders in women [1]. The syndrome is defined, after excluding other disorders which might present in a similar fashion, by occurrence of two of the following three features: i) oligo- or anovulation, ii) clinical and/or biochemical signs of hyperandrogenism, and iii) polycystic ovaries [2].

Severe insulin resistance characterises up to 90% of women with PCOS beyond that predicted by their body mass index (BMI) [2]–[4]. Indeed, even lean women with PCOS have elevated insulin resistance compared with BMI-matched controls [5].

As a consequence of highly common severe insulin resistance, the risk of impaired glucose tolerance (IGT) and type 2 diabetes mellitus (T2DM) in patients with PCOS significantly exceeds estimates of diabetes in the normal population [6]. Nevertheless, progression from insulin resistance to diabetes requires impaired β-cell function.

As regards insulin secretion, many studies have examined β-cell function in PCOS, but thus far, the results are equivocal. Studies have shown that there is a defect in the glucose-stimulated insulin secretion [7]–[9], but other studies have found that insulin response is enhanced in women with PCOS, likely as compensation for the peripheral insulin resistance [10]–[12].

Excess body weight in women with PCOS may accelerate progression toward diabetes by exacerbating both insulin resistance and inappropriate insulin-response. In a longitudinal study of 25 women with PCOS, the BMI of those whose glucose tolerance deteriorated was significantly higher than that of the group whose glucose tolerance improved or remained stable [13].

It is, thus, likely that obesity acts in concert with the intrinsic and apparently distinctive defects in insulin action of women with PCOS to enhance the risk of diabetes. However, no data have been provided on the effect of BMI on the compensatory insulin response, particularly in patients with a normal body weight.

Aim of the present investigation was to assess insulin resistance and compensatory insulin secretion by means of the oral glucose tolerance test (OGTT) in a sample of young PCOS women as compared to a large sample of age and body mass index matched controls. All cases and controls had normal glucose tolerance (NGT) and their BMI ranged from normality to severe obesity.

## Methods

A total of 69 women with clinical and hormonal evidence of PCOS were recruited at the Department of Internal Medicine, Catholic School of Medicine in Rome. All women had a standard oral glucose tolerance test (OGTT) with measurement of glucose, insulin and c-peptide levels at every 30 min time-point. They had normal fasting glucose (<5.6 mmol/l) and glucose tolerance (≤7.8 mmol/l at 2 h) [14].

PCOS was diagnosed in presence of at least two out of the three diagnostic criteria established by the revised 2003 Rotterdam European Society for Human Reproduction/American Society of Reproductive Medicine PCOS Consensus Workshop Group: i) oligo- and/or anovulation, ii) clinical and/or biochemical signs of hyperandrogenism, and iii) polycystic ovaries [2].

A pregnancy test was ordered on all subjects and none was found to be pregnant. The amount of excess terminal hair growth was first assessed using the modified Ferriman-Gallwey (mF-G) method, scoring the presence of terminal hairs over nine body areas (*i.e.* upper lip, chin, chest, upper and lower abdomen, thighs, upper and lower back, and upper arms) from 0 to 4. To maximize the accuracy of hirsutism scoring, subjects were examined by two physicians (EA & GM). Hirsutism was diagnosed by the presence of mF-G score ≥6 [15].

The presence of acne was also recorded, although no specific scoring system was used.

The study was performed in the early follicular phase (*i.e.* between d 2 and 5 after the last menstrual period). In oligo- or amenorrheic patients, the last menstrual period was either spontaneous or induced by the administration of didrogesterone (10 mg/d for 7 d).

Other endocrinopathies were ruled out by measuring thyroid stimulating hormone, basal serum 17-hydroxyprogesterone, prolactin, and 24 hour urine free cortisol levels. Exclusion criteria included age>50y, known cardiovascular disease, immune and non immune thyroid disease, neoplasm, current smoking, impaired glucose tolerance or diabetes, hypertension (blood pressure, BP, >135/85 mmHg), liver dysfunction, and renal impairment. None of the patients had taken any of the following medications for at least 6 months prior to enrolment in the study: hormonal contraceptives, glucocorticoids, ovulation induction agents, antidiabetic or antiobesity drugs, estrogenic, antiandrogenic, or antihypertensive medications.

Healthy normal-weight volunteers were recruited among medical students, young physicians, and nurses as normal-weight controls. Overweight/obese subjects with no evidence PCOS were recruited from those at their first referral to the Department of Internal Medicine at the Catholic University or the Research Unit for Multifactorial Disease at the Bambino Gesù Hospital for overweight/obesity. They were all free of medication despite severe obesity.

Most of the controls have been enrolled in previous studies [16]–[19] and were women aged 16–49 y on free diet, with regular menses, no clinical signs of hyperandrogenism (either hirsutism, acne or alopecia) or ultrasound evidence of polycystic ovaries; no systemic/endocrine disease including thyroid disease, no use of medication (including lipid lowering drugs, antihypertensive and insulin sensitizer agents or any medication able to interfere with glucose metabolism), no impaired fasting glucose or glucose intolerance. In all cases and controls, overweight was defined as a BMI ≥25.0 kg/m^2^ and obesity as a BMI ≥30 kg/m^2^.

The study was approved by the Institutional Review Board at the Catholic School of Medicine. It was conducted in accordance with the Declaration of Helsinki for research in human beings. All patients provided written informed consent prior to study participation. For adolescent girls, written full informed consent was obtained from parents/tutors and written assent absence from patient.

### Oral Glucose Tolerance Test and Insulin Metabolism

Blood samples were obtained at baseline and at 30-min intervals for 2 h for measurement of glucose, insulin and c-peptide after ingestion of the glucose load. Insulin resistance was estimated by the homeostasis model of insulin resistance (HOMA-IR; www.dtu.ox.ac.uk/homa) and the Whole-Body Insulin Sensitivity Index (WBISI) [20). Area under the glucose and the insulin curves were calculated by using the trapezoidal curve. The insulin secretion-sensitivity index-2 (ISSI-2) was calculated as the ratio of the area-under-the-insulin-curve to the area-under-the-glucose curve, multiplied by the Matsuda index [21]. β-cell function was assessed from the OGTT using the C-peptide deconvolution method [22] which provides estimation of both fasting and total insulin secretion and normalized for the body surface area. Total insulin secretion was normalized for a value of WBISI of 5 in order to allow the comparison of insulin secretion between cases and controls with different degree of insulin sensitivity.

### Analytical Methods

Serum concentrations of FSH, LH, estradiol (E2), testosteron (T), androstenedione (A), dehydroepiandrosterone sulphate (DHEAS), 17-hydroxyprogesterone (17-OHP), and Sex Hormone Binding Globulin (SHBG) were all assayed by commercially available radioimmunoassays (RIA; Radim, Pomezia, and Ares Serono, Milan, Italy). Samples were immediately processed in a refrigerated centrifuge and stored at −20°C until assay. The intra-assay and inter-assay coefficients of variation obtained were ≤9% for all variables. The free androgen index (FAI) was calculated by the formula: (total T/SHBG)×100. Serum AMH concentrations were determined by enzyme immunoassay (Immunotech–Beckman-Coulter, Fullerton, CA).

Serum glucose, insulin and c-peptide were analyzed at the Catholic University in all subjects. Glucose was measured by the glucose oxidase method (Beckman, Fullerton, CA). Insulin was assayed by microparticle enzyme immunoassay (MEIA; Abbott, Pasadena, CA) with a sensitivity of 1 μU/ml and an intra-assay coefficient of variation (CV) of 6.6%. C-peptide was assayed by radioimmunoassay (MYRIA; Technogenetics, Milan, Italy); this assay has a minimal detectable concentration of 17 pmol/l and intra-assay and interassay CVs of 3.3–5.7 and 4.6–5.3, respectively. Triglycerides, total and high-density lipoprotein (HDL) cholesterol were measured by using commercial methods (ADVIA 2400 Chemistry System, Siemens Healthcare Diagnostic, and Deerfield, IL).

### Statistical Analysis and Data Analysis

Continuous data are reported as means and standard deviations, with categorical data as counts and percentages. The Mann Whitney U-test and the nonparametric Spearman correlation coefficient were used for comparisons and correlations. The ANOVA analysis was used for comparisons among normal-weight, overweight and obese cases.

The *p* value was set as statistically significant at p<0.05.

Data analysis was performed by using SPSS statistical software (SPSS V17.0, Inc., Chicago, IL).

## Results

Patients were 69 young women (age 16–49 y, BMI 16.1–53.3 kg/m^2^); controls were 479 women of matched age (25.7±3.7 y). Among cases, 29 women were normal-weight, 24 overweight and 16 were obese patients. Controls were 271 normal-weight, 151 overweight and 57 obese women. **Tables 1** illustrates anthropometrics, laboratory parameters, and values of insulin metabolism in cases and controls according to their obesity status. In PCOS patients, HOMA-IR, WBISI, fasting and total insulin secretion, ISSI-2 were all statistically different respect with values observed in controls belonging to the different BMI groups.

**Table 1:** Anthropometrics, laboratory and insulin metabolism related parameters of controls and PCOS patients according to the obesity status.

	Normal weight women			Overweight women			Obese women		
	Controls	PCOS	P	Controls	PCOS	p	Controls	PCOS	p
**N**	271	29		151	24		57	16	
**Age (years)**	25.8±3.4	25.2±4.7	0.09	25.5±3.52	25.1±6.21	0.2	25.3±5.13	28.75±8.5	0.1
**BMI (kg/m^2^)**	21.5±1.8	21.1±1.8	0.3	26.5±1.7	26.6±1.7	0.8	34.3±4.3	37.4±6.6	0.06
**Waist circumference (cm)**	70.6±6.7	73.1±6.9	0.2	85.2±8.6	90.4±9.6	0.08	97.6±8.6	107.5±12.4	0.01
**Total cholesterol (mmol/l)**	4.23±0.6	4.53±0.8	0.03	4.44±0.8	4.61±0.8	0.3	4.66±0.83	4.82±0.93	0.6
**HDL-cholesterol (mmol/l)**	1.67±0.37	1.64±0.28	0.7	1.47±0.33	1.44±0.39	0.9	1.36±0.34	1.24±0.21	0.3
**LDL-cholesterol (mmol/l)**	2.32±0.63	2.5±0.72	0.2	2.72±0.76	2.53±0.82	0.3	2.89±0.86	3.09±0.86	0.2
**Triglycerides (mmol/l)**	0.62±0.35	0.78±0.34	<0.0001	0.93±0.47	1.03±0.73	0.9	1.03±0.45	1.22±0.63	0.3
**Fasting glucose (mmol/l)**	4.73±0.51	4.8±0.36	0.7	4.94±0.48	4.91±0.47	0.7	4.90±0.45	5.13±0.59	0.4
**Fasting Insulin (μUI/ml)**	4.38±2.23	9.41±4.08	<0.0001	6.59±6.58	11.13±3.67	<0.0001	12.26±8.25	20.63±9.61	<0.0001
**2 hour glucose (mmol/l)**	5.11±0.96	5.22±1.15	0.6	5.37±1.05	5.36±1.17	0.9	5.79±1.21	5.84±1.14	0.9
**HOMA-IR**	0.91±0.49	2.01±0.89	<0.0001	1.36±0.65	2.43±0.86	<0.0001	2.19±1.09	4.77±2.62	<0.0001
**WBISI**	12.8±5.9	6.8±2.95	<0.0001	9.09±4.16	5.31±1.80	<0.0001	5.73±3.24	3.14±1.76	<0.0001
**Fasting insulin secretion (pmol min^−1^m^−2^)**	61.55±22.2	82.1±26.8	<0.0001	74.29±25.7	94.92±28.0	0.001	114.9±37.7	157.8±51.2	<0.0001
**Total insulin secretion (nmol m^−2^)**	37.9±12.1	60±23.6	<0.0001	39.1±11.8	53.95±14	<0.0001	52±16.5	69.8±21.6	<0.0001
**AUC insulin (μUI/ml min^−1^)**	50.6±36.9	197±109.1	<0.0001	54.9±22.3	240.5±116.9	<0.0001	96.15±62.3	381±227.6	<0.0001
**Total Insulin Secretion@5WBISI (nmol m^−2^)**	0.80±0.72	2.48±2.45	<0.0001	1.14±0.85	2.36±1.16	<0.0001	2.44±1.68	6.08±4.04	<0.0001
**ISSI-2**	2.71±1.1	2.37±1	0.4	2.36±1.03	2.14±0.70	0.3	1.98±0.79	1.82±0.63	0.7

Data are expressed as mean ± SD. *P* refers to statistical significance at the Mann-Whitney U test within groups of normal-weight, overweight and obese women. BMI, body Mass Index; HDL, High density lipoprotein; HOMA-IR, Homeostasis Model Assessment of Insulin Resistance; LDL, Low density lipoprotein; WBISI, Whole Body Insulin Sensitivity Index; ISSI-2, Insulin Secretion-Sensitivity Index-2; Total Insulin, Total insulin secretion at a WBISI of 5.

Regards cases, 66 patients (95.6%) presented with hirsutism, which was equally distributed across classes of BMI; 38 (55%) had acne and 7 (10%) alopecia. The mean m-FG was 14.2±5.3. Oligo/anovulation was diagnosed in 48 cases (69.6%) and ultrasound evidence of polycystic ovaries was observed in 39 cases (56.3%). At the ANOVA, obese cases were the oldest (p = 0.02), presented with the highest concentrations of SHBG (p = 0.012); fasting insulin (p<0.0001), FAI (p = 0.023); the worst lipid profile (HDL cholesterol, p<0.0001; LDL-cholesterol, p = 0.007; and triglycerides, p = 0.009), insulin action (HOMA-IR and WBISI, p<0.00001 for both); and the highest fasting insulin secretion (p<0.0001).

**Figure 1:**
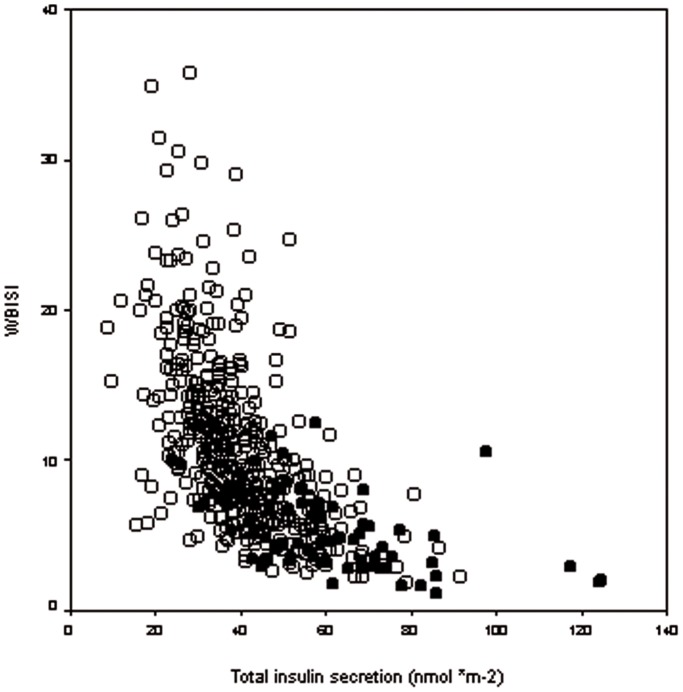
Relationship between insulin sensitivity and secretion in cases and controls. Scatter plot of the relationship between whole body insulin sensitivity (WBISI) and Total insulin secretion in controls (empty circles) and patients with PCOS (filled circles).

**Figure 1** shows the relationship between WBISI and total insulin secretion in cases and controls.

**Figure 2:**
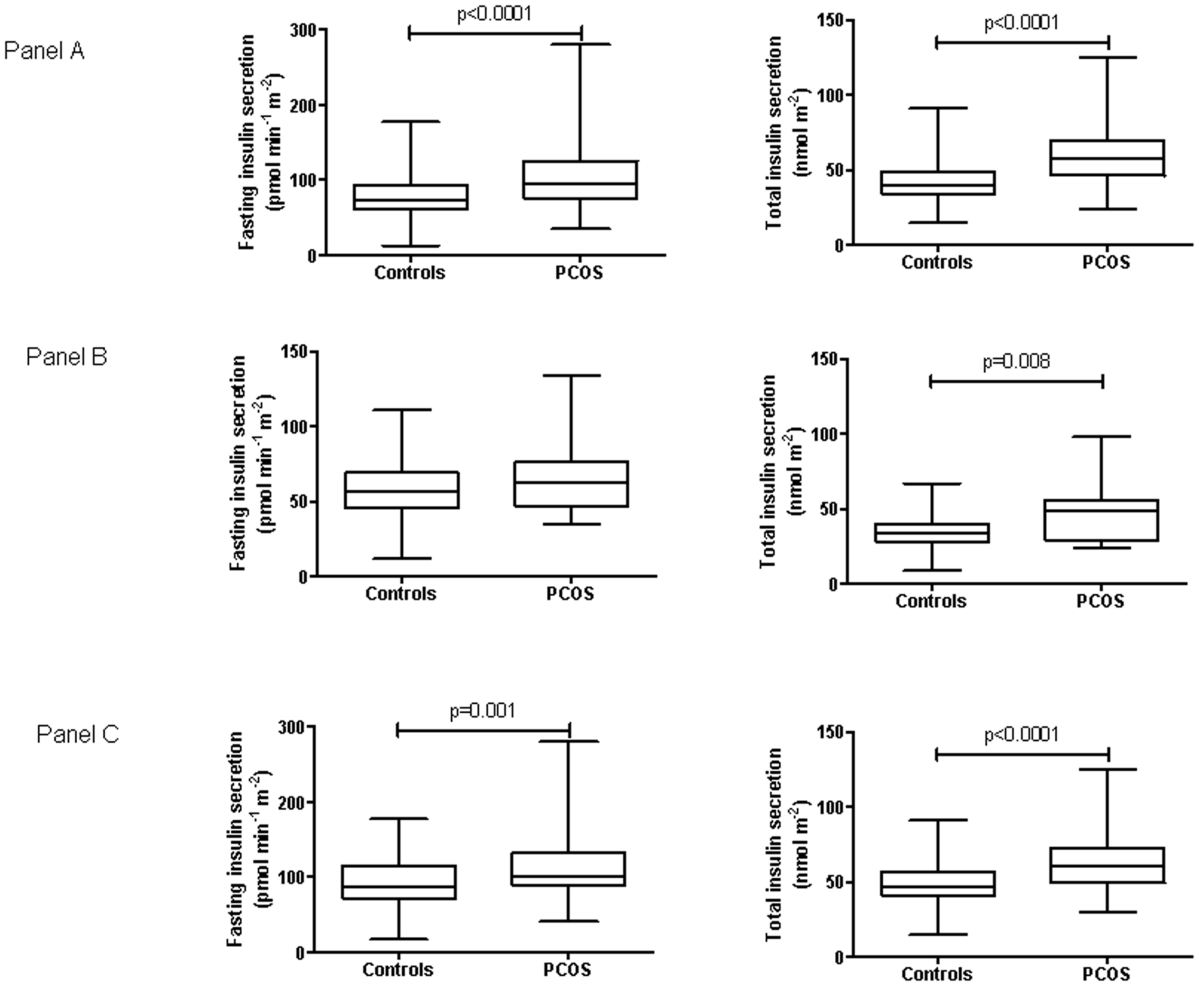
Insulin secretion in insulin sensitive and resistant cases and controls. Fasting and total insulin secretion (box and whiskers, min to max) in control and PCOS women having insulin resistance defined as HOMA-IR ≥1.26 (panel A); insulin sensitivity defined as WBISI ≥7.91 (panel B) and insulin resistance as WBISI<7.91 (panel C).

To further assess whether insulin hypersecretion in PCOS patients results from increased insulin resistance, control and PCOS women were successively defined as insulin sensitive or resistant by using tertiles of both HOMA-IR and WBISI in controls (insulin resistance as HOMA-IR≥1.26 and WBISI<7.91). Accordingly, 98.6% of PCOS patients were resistant having HOMA-IR≥1.26 and 81.2% with WBISI <7.91. **Figure 2** shows in control and PCOS women values of fasting and total insulin secretion in the groups of women insulin resistant according to the HOMA-IR index (panel A); in insulin sensitive (panel B) and insulin resistant women (panel C) according to the values of WBISI.

Mean concentrations of androgens and sex hormones were as it follows: A_4_ 227.4±36.2 ng/dl; AMH 7.40±3.2 ng/ml; T 6.65±4.43 ng/dl; DHEAs 249.7±113.4 μg/dl; E_2_ 99.4±68.3 pg/ml; FAI 6.65±7.4 ng/100 ml; LH 5.4±3.08 IU/l; FSH 4.45±1.78 IU/l; LH/FSH 1.24±0.56; SHBG 45.15±36.2 nmol/l.

### Correlation Analysis

In cases, fasting insulin secretion and WBISI were significantly correlated with BMI (*r_o_* = 0.571; p<0.0001 and *r_o_* = −0.520; p<0.0001, respectively) and waist circumference (*r_o_* = 0.687; p<0.0001, and *r_o_* = −0.590; p<0.0001). **Table 2** reports correlation coefficients of androgens and sex hormone with BMI, HOMA-IR, WBISI, fasting and total insulin secretion, ISSI-2.

**Table 2:** Coefficients of correlation in patients with PCOS.

	Body weight	HOMA-IR	WBISI	Fasting insulin secretion	Total Insulin secretion	ISSI-2
**A_4_**	0.131 *P* = 0.302	**0.377 ** ***p = *** **0.006**	−**0.395 ** ***p*** ** = 0.001**	**0.252 ** ***p*** ** = 0.045**	0.208 *p* = 0.09	0.006 *p = 0.962*
**AMH**	−0.231 *P = *0.1	−0.137 *p = 0.334*	0.142 *p = 0.317*	−0.157 *p* = 0.266	−0.055 *p* = 0.701	0.006 *p = 0.966*
**DHEAS**	0.183 *p = *0.13	0.168 *p = 0.174*	−**0.256 ** ***p*** ** = 0.037**	0.122 *p* = 0.325	0.189 *p* = 125	0.140 *p = 0.259*
**E_2_**	−0.037 *p = *0.771	−0.106 *p = 0.399*	0.022 *p* = 0.859	−0.90 *p* = 0.473	0.084 *p* = 0.504	−0.10 *p = 0.934*
**FAI**	**0.472 ** ***P*** **<0.0001**	**0.503 ** ***p<0.0001***	−**0.606 ** ***p*** **<0.0001**	**0.494 ** ***p*** **<0.0001**	**0.459 ** ***p*** **<0.0001**	−0.075 *p = 0.546*
**FSH**	−0.068 *p = *0.623	**0.269 ** ***p = 0.047***	−0.257 *p* = 0.06	0.150 *p* = 0.447	0.004 *p* = 0.974	−0.281 *p = 0.083*
**LH**	0.122 *p = *0.623	**0.322 ** ***p = 0.016***	−**0.298 ** ***p*** ** = 0.03**	**0.378 ** ***p*** ** = 0.004**	0.244 *p = *0.07	−0.234 *p = 0.03*
**LH/FSH**	0.124 *p = *0.377	0.173 *p = 0.216*	−0.175 *p = 0.209*	**0.273 ** ***p = 0.048***	**0.366 ** ***p = 0.007***	−0.109 *p = 0.439*
**SHBG**	−**0.546 ** ***P*** **<0.0001**	−**0.555 ** ***p<0.0001***	**0.566 ** ***p*** **<0.0001**	−**0.574 ** ***p*** **<0.0001**	**−0.359 ** ***p*** ** = 0.003**	0.034 *p = 0.784*
**T**	**0.472 ** ***P<0.0001***	**0.503 ** ***p<0.0001***	**−0.606 ** ***p<0.0001***	**0.494 ** ***p<0.0001***	**0.459 ** ***p<0.0001***	−0.75 *p = 0.546*

A4, Androstenedione; AMH, Anti-Mullerian Hormone; BMI, body Mass Index;

CBG, Cortisol Binding Globulin; DHEAS, Dehydroepiandrosterone sulfate; E2, 17β-estradiol;

HOMA-IR, Homeostasis Model Assessment of Insulin Resistance; LH, Luteinizing Hormone;

FAI, Free androgen index; FSH, follicle-stimulating hormone; T, testosterone; WBISI, Whole Body Insulin Sensitivity Index; ISSI-2, Insulin.

In controls, fasting and total insulin secretion were significantly correlated with BMI (*r_o_* = 0.443; p<0.0001; *r_o_* = 0.230; p<0.0001) and waist circumference (*r_o_* = 0.429; p<0.0001; *r_o_* = 0.175; p<0.0001). WBISI was correlated with BMI (*r_o_* = −0.482; p<0.0001) and waist (*r_o_* = 0.477; p<0.0001). ISSI-2 was correlated with waist (*r_o_* = 0.776; p<0.0001).

Furthermore, multivariate analysis was performed. Fasting secretion was predicted (r^2^ = 0.46; p<0.0001) by having PCOS (coefficient 17; 95%CI 10.5–2.35), being obese (coefficient 15.7; 95%CI 12.3–19), and insulin resistant (coefficient 26.9; 95%CI 22–31.8); total insulin secretion by having PCOS (coefficient 14; 95%CI 10.8–17.4), and being insulin resistant. (coefficient 13.6; 95%CI 11.1–16).

## Discussion

Young women with polycystic ovary syndrome but normal glucose tolerance are able to compensate for severe insulin resistance with enhanced β-cell function. They are less insulin sensitive but able to release much more insulin following the glucose ingestion than age and BMI matched controls **(** **Figure 1** **)**. Indeed, insulin secretion was normalized for a fixed value of WBISI to take into account differences in insulin sensitivity between PCOS patients and controls and the former hyper-secreted insulin in comparison with insulin-resistance matched control women.

Among the three groups of patients with PCOS differing by the obesity status, lipid profile and insulin action worsened and β-cell insulin release increased significantly with the increasing BMI (**Table 1**). In our series, the use of a glucose disposition index (the ISSI-2) provides an estimate of β-cell functionality relative to the prevailing level of insulin resistance. Moreover, we normalised total insulin secretion by the fixed value of insulin sensitivity of 5 to compare secretion among cases and controls with different BMI. Furthermore, when control and PCOS women were divided as insulin sensitive or resistant according to tertiles of HOMA-IR or WBISI, PCOS patients showed still higher fasting and total insulin secretion (**Figure 2**).

Past studies that investigated either insulin action by using the euglycemic hyperinsulinemic clamp, which is considered the gold-standard technique to estimate insulin sensitivity, or the intravenous glucose tolerance test (IVGTT) and β-cell function by the c-peptide deconvolution method, provided divergent results.

Indeed, about half of the past clamp studies found preserved insulin sensitivity in normal-weight women with PCOS when compared with weight-matched controls [10]–[11], [23]–[26], but some other studies reported some degree of insulin resistance [4], [13], [27]–[28]. By using the IVGTT, Gennarelli *et al* [25] found in women with PCOS a significant reduction of the glucose effectiveness, *i.e.* the proportion of glucose uptake independent from insulin activity, while insulin sensitivity, i.e. the proportion of glucose uptake dependent of insulin was normal. As reminded by the authors, the clamp cannot discriminate between insulin dependent and insulin independent glucose uptake, but in normal individuals between 30 and 50% of glucose disposal after an oral glucose load depends on glucose effectiveness [29] which represents the major contributor to glucose disappearance in states of severe insulin resistance [30]. Hence, reduced glucose effectiveness in patients with PCOS would support lower insulin sensitivity following the glucose load as we observed in the present investigation.

Some studies that carefully assessed β-cell function have claimed dysfunctional insulin secretion [7], [31]–[32]. Our data do not support dysfunctional insulin secretion, at least in glucose normotolerant cases, as the latter women release much more insulin than age, BMI and insulin resistance-matched controls following the glucose load. Findings from our study are in keeping with results of a previous study which found in PCOS women higher basal and 24 h insulin concentration, reduced insulin clearance and increased secretion by analysis of 24 hour profiles of insulin and c-peptide and hyperglycaemic clamp [31]. While basal insulin secretion was significantly increased as compared to secretion in controls, their incremental insulin response to a meal was significantly reduced. The reduction in the post-meal response resulted from a reduction in the relative amplitude of meal-related secretory pulses rather than from a reduction in the number of pulses [31]. In our series we did not estimate secretory pulses, but both absolute under the insulin curve and incremental areas were significantly greater in patients with PCOS (**Table 1**). At the IVGTT, Hermann *et al* [32] found that the ability of the β-cell to respond to oscillations in plasma glucose is impaired in women with PCOS. Arslanian *et al* [8] studied NGT and IGT PCOS adolescents. They observed not only reduced insulin sensitivity at the euglycemic clamp, but reduced first phase insulin secretion and glucose disposition index and increased hepatic glucose production by the hyperglycaemic clamp but limited to IGT patients. On the contrary, Gennarelli *et al* [25] and Holte *et al* [10] ^.^ found no significant difference in the acute (“first-phase”) insulin response to intravenous glucose administration.

On purpose, we excluded from our investigation women with IGT, who, for sure would present with increased insulin resistance, defective β-cell function and, therefore, reduced glucose disposition index to focus, on the contrary, on NGT women with various BMI, from normal weight to excess weight, to answer the question whether β-cell function is dysfunctional or, conversely, women with PCOS have insulin hypersecretion and to look at the impact of excessive body weight on insulin secretion.

Major drawbacks of our study are due to the cross-sectional design of the study, the small size-sample of cases, the lack of information on body composition and family history on diabetes as it is well accepted that the presence of family history of T2DM in women with PCOS increases the likelihood of inappropriate β-cell compensation for the degree of insulin resistance [32].

If the enrolment exclusively of cases with normal glucose tolerance in our series does not allow comparing our findings with other researchers’ results as they enrolled mostly women with altered glucose tolerance, this choice, however, represents the strength of our study. The lesson from PCOS patients with normal glucose tolerance when considering their high risk of diabetes may be that the β-cell of a woman with PCOS is very early challenged by enhanced insulin resistance and obesity exacerbates this load. The β-cell response to such a challenge may result defective in the long-term in those women who are genetically prone to diabetes, but in women who maintain a normal glucose tolerance it seems to be significantly increased.

Regards to the role of excessive androgens in the natural progression from NGT to diabetes in women with PCOS, our findings are confirmatory of the association between A4, free testosterone and SHBG with enhanced insulin resistance [32], but they also suggest that hyperandrogenemia may affect also insulin secretion as FAI and concentration of SHBG were correlated with insulin secretion (**Table 2**). Unfortunately, as the cross-sectional nature of the present study, we could not rule out whether such effect is direct on the β-cell or indirect through increased insulin resistance and whether β-cell becomes impaired with aging.

In conclusion, PCOS patients with normal glucose tolerance are more insulin resistant than controls, their insulin secretion increases in parallel with raised insulin secretion, but, more importantly, they are able to secrete much more insulin than insulin sensitivity matched peers. As expected, increasing BMI exerts a major effect on both insulin resistance and β-cell insulin response, but high levels of androgens seem to affect both sites, i.e. insulin action and insulin secretion, of the glucose metabolism.

## References

[1] Randeva HS, Tan BK, Weickert MO, Lois K, Nestler JE, et al (2012) Cardiometabolic Aspects of the Polycystic Ovary Syndrome. Endocr Rev. Oct 33(5): 812–41. 10.1210/er.2012-1003 PMC3461136 22829562

[2] The Rotterdam ESHRE/ASRM-Sponsored PCOS consensus workshop group Revised 2003 consensus on diagnostic criteria and long-term health risks related to polycystic ovary syndrome (PCOS) (2003) Human Reproduction. 19: 41–47. 10.1093/humrep/deh098 14688154

[3] Carmina E, Lobo RA (1999) Polycystic ovary syndrome (PCOS): arguably the most common endocrinopathy is associated with significant morbidity in women. JClin Endocrinol Metab. 84: 1897–1899. 10.1210/jcem.84.6.5803 10372683

[4] Dunaif A, Segal KR, Futterweit W, Dobrjansky A (1989) Profound peripheral insulin resistance, independent of obesity, in polycystic ovary syndrome. Diabetes. 38: 1165–1174. 10.2337/diab.38.9.1165 2670645

[5] Dunaif A (1999) Insulin action in the polycystic ovary syndrome. Endocrinol Metab Clin North Am. 28: 341–359. 10.1016/s0889-8529(05)70073-6 10352922

[6] Legro RS, Kunselman AR, Dunaif A (2001) Prevalence and predictors of dyslipidemia in women with polycystic ovary syndrome. Am J Med. 111: 607–613. 10.1016/s0002-9343(01)00948-2 11755503

[7] Dunaif A, Finegood DT (1996) Beta-cell dysfunction independent of obesity and glucose intolerance in the polycystic ovary syndrome. J Clin Endocrinol Metab. 81: 942–947. 10.1210/jcem.81.3.8772555 8772555

[8] Arslanian SA, Lewy VD, Danadian K (2001) Glucose intolerance in obese adolescents with polycystic ovary syndrome: roles of insulin resistance and beta-cell dysfunction and risk of cardiovascular disease. J Clin Endocrinol Metab. 86: 66–71. 10.1210/jcem.86.1.7123 11231980

[9] Ehrmann DA, Barnes RB, Rosenfield RL, Cavaghan MK, Imperial J (1999) Prevalence of impaired glucose tolerance and diabetes in women with polycystic ovary syndrome. Diabetes Care. 22: 141–146. 10.2337/diacare.22.1.141 10333916

[10] Holte J, Bergh T, Berne C, Wide L, Lithell H (1995) Restored insulin sensitivity but persistently increased early insulin secretion after weight loss in obese women with polycystic ovary syndrome. J Clin Endocrinol Metab. 80: 2586–2593. 10.1210/jcem.80.9.7673399 7673399

[11] Morin-Papunen LC, Vauhkonen I, Koivunen RM, Ruokonen A, Tapanainen JS (2000) Insulin sensitivity, insulin secretion, and metabolic and hormonal parameters in healthy women and women with polycystic ovarian syndrome. Hum Reprod. 15: 1266–1274. 10.1093/humrep/15.6.1266 10831553

[12] Vrbikova J, Grimmichova T, Dvorakova K, Hill M, Stanicka S, et al (2008) Family history of diabetes mellitus determines insulin sensitivity and beta cell function in polycystic ovary syndrome. Physiol Res. 57: 547–553. 10.33549/physiolres.931275 17705674

[13] Dunaif A, Segal KR, Shelley DR, Green G, Dobrjansky A, et al (1992) Evidence for distinctive and intrinsic defects in insulin action in polycystic ovary syndrome. Diabetes. 41: 1257–1266. 10.2337/diab.41.10.1257 1397698

[14] American Diabetes Association (2004) Diagnosis and classification of diabetes mellitus. Diabetes Care. 27 Suppl 1S5–S10. 10.2337/diacare.27.2007.s5 14693921

[15] Ferriman DM, Gallwey JD (1961) Clinical assessment of body hair growth in women. J Clin Endocrinol. 21: 1440–1447. 10.1210/jcem-21-11-1440 13892577

[16] Mingrone G, Manco M, Mora ME, Guidone C, Iaconelli A, et al (2008) Influence of maternal obesity on insulin sensitivity and secretion in offspring. Diabetes Care. Sep 31(9): 1872–6. 10.2337/dc08-0432 PMC2518362 18535193

[17] Manco M, Panunzi S, Macfarlane DP, Golay A, Melander O, et al (2010) Relationship between Insulin Sensitivity and Cardiovascular Risk (RISC) Consortium. One-hour plasma glucose identifies insulin resistance and beta-cell dysfunction in individuals with normal glucose tolerance: cross-sectional data from the Relationship between Insulin Sensitivity and Cardiovascular Risk (RISC) study. Diabetes Care. 33(9): 2090–7. 10.2337/dc09-2261 PMC2928370 20805281

[18] Nolfe G, Spreghini MR, Sforza RW, Morino G, Manco M (2012) Beyond the morphology of the glucose curve following an oral glucose tolerance test in obese youth. Eur J Endocrinol. 166(1): 107–14. 10.1530/EJE-11-0827 22009494

[19] Manco M, Miraglia Del Giudice E, Spreghini MR, Cappa M, Perrone L, et al (2012) 1-Hour plasma glucose in obese youth. 49(6): 435–43. 10.1007/s00592-012-0384-3 22391936

[20] Matsuda M, DeFronzo RA (1999) Insulin sensitivity indices obtained from oral glucose tolerance testing: comparison with the euglycemic insulin clamp. Diabetes Care 22 1462–1470. 10.2337/diacare.22.9.1462 10480510

[21] Retnakaran R, Qi Y, Goran MI, Hamilton JK (2011) Evaluation of proposed oral disposition index measures in relation to the actual disposition index. Eur J Endocrinol. Aug 165(2): 323–30. 10.1111/j.1464-5491.2009.02841.x 20002470

[22] Van Cauter E, Mestrez F, Sturis J, Polonsky KS (1992) Estimation of insulin secretion rates from C-peptide levels. Comparison of individual and standard kinetic parameters for C-peptide clearance. Diabetes. Mar 41(3): 368–77. 10.2337/diab.41.3.368 1551497

[23] Ovesen P, Moller J, Ingerslev HJ, Jorgensen JO, Mengel A, et al (1993) Normal basal and insulin-stimulated fuel metabolism in lean women with the polycystic ovary syndrome. J Clin Endocrinol Metab. 77: 1636–1640. 10.1210/jcem.77.6.8263152 8263152

[24] Ciampelli M, Fulghesu AM, Cucinelli F, Pavone V, Caruso A, et al (1997) Heterogeneity in β cell activity, hepatic insulin clearance and peripheral insulin sensitivity in women with polycystic ovary syndrome. Hum Reprod. 12: 1897–1901. 10.1093/humrep/12.9.1897 9363702

[25] Gennarelli G, Holte J, Berglund L, Berne C, Massobrio M, et al (2000) Prediction models for insulin resistance in the polycystic ovary syndrome. Hum Reprod. 15: 2098–2102. 10.1093/humrep/15.10.2098 11006180

[26] Cibula D, Sindelka G, Hill M, Fanta M, Skrha J, et al (2002) Insulin sensitivity in non-obese women with polycystic ovary syndrome during treatment with oral with oral contraceptives containing low-androgenic progestin. Human Reprod. Jan 17(1): 76–82. 10.1093/humrep/17.1.76 11756365

[27] Toprak S, Yonem A, Cakir B, Guler S, Azal O, et al (2001) Insulin resistance in nonobese patients with polycystic ovary syndrome. Horm Res. 55: 65–70. 10.1159/000049972 11509861

[28] Diamanti Kandarakis E, Mitrakou A, Hennes MM, Platanissiotis D, Kaklas N, et al (1995) Insulin sensitivity and antiandrogenic therapy in women with polycystic ovary syndrome. Metabolism. 44: 525–531. 10.1016/0026-0495(95)90062-4 7723677

[29] Best JD, Kahn SE, Ader M, Watanabe RM, Ta-Chen N, et al (1996) Role of glucose effectiveness in the determination of glucose tolerance. Diabetes Care. 19: 1018–1030. 10.2337/diacare.19.9.1018 8875104

[30] Ahrèn B, Pacini G (1998) Age-related reduction in glucose elimination is accompanied by reduced glucose effectiveness and increased hepatic insulin extraction in man. J Clin Endocrinol Metab. 83: 3350–3356. 10.1210/jcem.83.9.5107 9745453

[31] O’Meara NM, Blackman JD, Ehrmann DA, Barnes RB, Jaspan JB, et al (1993) Defects in beta-cell function in functional ovarian hyperandrogenism. J Clin Endocrinol Metab. 76(5): 1241–7. 10.1210/jcem.76.5.8496316 8496316

[32] Ehrmann DA, Sturis J, Byrne MM, Karrison T, Rosenfield RL, et al (1995) Insulin secretory defects in polycystic ovary syndrome. Relationship to insulin sensitivity and family history of non-insulin-dependent diabetes mellitus. J Clin Invest. 96: 520–527. 10.1172/JCI118064 PMC185226 7615824

